# Prevalence and Clinical Significance of* Streptococcus dysgalactiae subspecies equisimilis* (Groups C or G Streptococci) Colonization in Pregnant Women: A Retrospective Cohort Study

**DOI:** 10.1155/2018/2321046

**Published:** 2018-06-03

**Authors:** M. Jaalama, O. Palomäki, R. Vuento, A. Jokinen, J. Uotila

**Affiliations:** ^1^Department of Obstetrics and Gynecology, Kanta-Häme Central Hospital, Hämeenlinna, Finland; ^2^Department of Obstetrics and Gynecology, Tampere University Hospital, Tampere, Finland; ^3^Department of Microbiology, Fimlab Laboratories Ltd., Tampere, Finland; ^4^Department of Obstetrics and Gynecology, Central Finland Health Care District, Jyväskylä, Finland; ^5^School of Medicine, University of Tampere, Finland

## Abstract

**Objectives:**

Little is known about the significance of Streptococcus G or C colonization in pregnant women. The objective of this study was to assess whether vaginal Streptococcus group G or C colonization detected in late pregnancy increases the infectious morbidity of the mother or newborn.

**Methods:**

A total of 15,114 rectovaginal cultures taken at 35–37 weeks of pregnancy were analyzed at Tampere University Hospital, Finland, between 2012 and 2014. From this laboratory data, all Streptococcus G or C-positive cultures were included to study maternal and neonatal infectious morbidity after delivery. This study population was compared to women with a positive Streptococcus B culture and to women with a negative culture.

**Results:**

The prevalence of Streptococcus G or C colonization was 2.9%. Significantly more postpartum endometritis was found in this study group. No association was found between colonization and neonatal bacteremia.

**Conclusions:**

Streptococcus G or C colonization is associated with postpartum endometritis. More research is needed to clarify if antibiotic prophylaxis is reasonable for this group during delivery.

## 1. Introduction

*Streptococcus dysgalactiae subspecies equisimilis* (SDSE, Streptococcus group C or G) belongs to the group of beta-hemolytic Streptococci. Groups C and G Streptococci are often also referred to as pyogenic Streptococci because they are genetically close to* Streptococcus pyogenes* [1]. Vandamme et al. [2] divided* Streptococcus dysgalactiae* into two subspecies: SDSE was proposed for human groups and* Streptococcus dysgalactiae subspecies dysgalactiae* (SDSD) for strains of animal origin. SDSE have been identified as part of the normal flora of the human respiratory, gastrointestinal, and female genital tracts. Thus, for many years colonization by these bacteria has been considered harmless. However, the pathogenicity of SDSE has now been better recognized. Recent epidemiological studies have shown that SDSE causes a variety of diseases similar to those caused by* Streptococcus pyogenes*. Skin and soft tissue infections range from superficial wound infections to severe invasive infections, such as necrotizing fasciitis and life-threatening Streptococcal toxic shock syndrome (STSS) and bacteremia [3–5]. In 2009, Broyles et al. [6] reported almost 500 invasive SDSE infections in a large population-based epidemiological study in North America. In this study, 64% of the patients were males and 87% of the patients had underlying diseases. The mortality rate to bacteremia was 12%, similar to that reported elsewhere [7, 8]. In Finland, a 2.7-fold rise in Streptococcus G or C bacteremia was found among adult patients during a ten-year period between 1995 and 2004. This research showed that a disruption of the cutaneous barrier was an important predisposing factor [9, 10]. In addition, an association has been found between bacteremia and underlying conditions such as alcoholism, diabetes mellitus, immunosuppression, and advanced age [6, 11, 12].

Although SDSE is increasingly recognized as an important human pathogen, there is only limited information about the clinical role of SDSE colonization in pregnant women. As there are several changes in the immune system of pregnant women, SDSE colonization could be potentially pathogenic for pregnant women and newborns. Lu et al. [13] have reported on a surgical-site infection due to SDSE after cesarean section in a patient whose genital tract was colonized with Streptococcus G. In another case report [14], an otherwise healthy nonimmunocompromised young adult woman at 17 weeks of gestation developed meningitis caused by SDSE. It was thought that Streptococcal meningitis was acquired via transient colonization of the nasopharynx followed by bacteremia and invasion of the central nervous system. Faix et al. [15] found group C Streptococci in the cerebrospinal fluid of a newborn infant whose mother had chorioamnionitis during the delivery. In 1985, Vartian et al. [11] reported four cases of puerperal sepsis among fifty-seven cases of group G Streptococcal infection. In these and in the majority of other reported cases of puerperal infections due to G Streptococci, patients recovered promptly after antibiotic therapy [12]. Despite the increasing use of antibiotics, Streptococcus B and Streptococci G and C have remained sensitive to penicillin and other beta-lactam antibiotics [16].

The aim of this study was to determine the proportion of SDSE colonization at 35−37 weeks of gestation from 15,144 rectovaginal swab cultures and to assess the rate of early maternal and neonatal postpartum infections associated with SDSE colonization compared to patients with a positive Streptococcus B culture or a negative culture.

## 2. Materials and Methods

Tampere University Hospital is the tertiary care hospital for the ca. 524 700 inhabitants of Pirkanmaa Health District. There are about 5300 deliveries at the hospital annually. Universal screening for group B Streptococci was started in the hospital's catchment area in 2012. Laboratory testing primarily analyzes the colonization of group B, but beta-hemolytic Streptococci from other Lancefield groups, including C and G, are also revealed. All cases of maternal SDSE colonization based on a rectovaginal culture between January 1, 2012, and December 31, 2014, were identified from the laboratory database. The cultures were taken at 35–37 weeks of gestation using one swab from distal part of vagina, perineal area, and anus. The swab was swept first in the vagina and then the same swab was swept along the perineal area to the anus.

After the exclusion of cases with multiple colonization by both Streptococcus B and either Streptococcus C or Streptococcus G, there were 421 Streptococcus G or C-positive cultures in the final analyses. For these cases, the next group B Streptococci (GBS) colonization and the next negative culture were selected from the database to form two control groups. Thus, the entire study material consisted of 1263 women, whose rectovaginal culture was either Streptococcus C or Streptococcus G only, Streptococcus B only or negative, and their 1277 newborns. The maternal characteristics and early maternal and neonatal infections after delivery of the three groups were compared. Neonatal and obstetrics data, including discharge diagnoses, infection register data, and laboratory files for any culture-positive cases, were retrieved from the hospital's computerized databases.

Streptococcus B-positive mothers received prophylactic antibiotic therapy, which at our medical center is routinely penicillin G administered intravenously every fourth hour during labor. The groups with negative cultures or SDSE colonization were instructed to receive antibiotics only if there were signs of clinical chorioamnionitis during the delivery: presence of fever, fetal tachycardia, or laboratory findings suggesting infection. The most common antibiotic treatment for infection was cefuroxime or penicillin G combined with metronidazole intravenously. Women undergoing cesarean delivery were routinely given intraoperative antibiotic prophylaxis, usually cefuroxime 1.5 g intravenously. In postpartum endometritis, the routine intravenous antibiotic treatment was cefuroxime 1.5 g and metronidazole 500 mg three times per day. The most frequently used oral antibiotics were cephalexin 500 mg and metronidazole 500 mg (three times per day for 7–10 days).

### 2.1. Laboratory Analysis

Specimens were inoculated onto selective screening plates for beta-hemolytic Streptococci (blood agar base with 5% sheep's blood and a colistin and oxolinic acid supplement (LabM, Lancashire, UK)). Selective plates were incubated in 5% carbon dioxide for 24 hours. The Lancefield serogroups of typical beta-hemolytic colonies from selective plates were defined by latex agglutination using the Streptex latex test system (Remel Europe Ltd., Dartford, UK). MALDI-TOF MS (bioMérieux) was used to confirm the identification of Streptococci

### 2.2. Statistical Analyses

Statistical analyses were conducted using SPSS software (IBM SPSS Statistics for Windows, Version 23.0). ANOVA or the Kruskall–Wallis test was used to test differences in mothers' age and body mass index (calculated as weight in kilograms divided by height in meters squared) between the three study groups (SDSE, Streptococcus B, and negative culture), depending on whether the data had a normal or skewed distribution. The chi-squared test or Fisher's exact test was used to test differences between the three groups for the categorical variables. A *p* value of <0.05 was considered statistically significant.

### 2.3. Ethical Approval

The study protocol was approved by the Pirkanmaa Hospital District's ethical committee (decision R15517S, 19th March 2015).

## 3. Results

**Figure 1:**
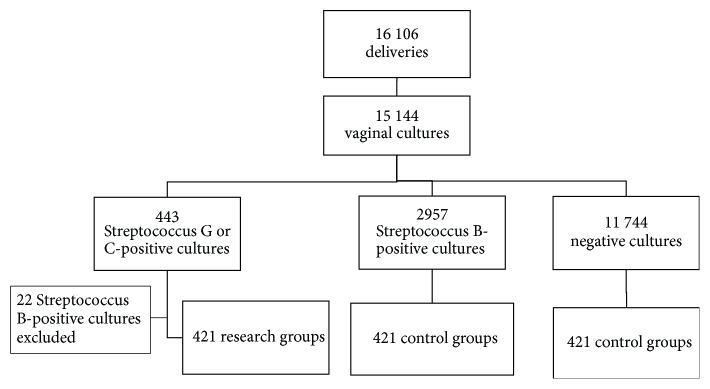
Flow diagram of the study participants (total *n* = 1263).

The swab cultures were taken from 94% of pregnant woman, producing 15,144 samples. Out of these, 443 (2.9% of all samples) were Streptococcus G or C-positive, 2957 (19.5% out of all samples) were Streptococcus B-positive, and 11, 744 (77,5%) were negative. There were 22 samples, in which both Streptococcus G or C and Streptococcus B were positive; these cases were excluded, and all other Streptococcus G or C-positive women remained as study group. From the other groups, similar amount of women were randomly selected into the two control groups. Selection of the study cases is shown in Figure 1. The clinical characteristics and demographic data are summarized in Table 1. There were no differences between the subgroups in the mean age, body mass index, or the cesarean section rate, but there were more postterm pregnancies in the Streptococcus G or C group.

Of the 1263 study subjects, a total of 35 maternal infections were diagnosed after delivery. The most common puerperal infection was postpartum endometritis. The incidences of any infections and endometritis in the SDSE colonization group were significantly higher than in the other two groups (5.7% versus 1.7% versus 1.0% for any puerperal infection in the groups for SDSE, Streptococcus B, or culture negative, respectively). There was no difference between the subgroups in the incidence of urinary tract infection, wound infections, or bacteremia, as shown in Table 2.

The 18 endometritis cases in the Streptococcus G or C group were further analyzed and the data are shown in Table 3. Commonly, the symptoms (foul smelling lochia, abdominal pain, and fever) started 3–10 days after vaginal delivery, and leukocytosis and a raised CRP value were present. 67% (12/18) of patients were given intravenous antibiotics, and the mean highest C-reactive protein (CRP) was 122 mg/L (range 8–267 mg/L). After 7–10 days of combined antibiotic treatment (cephalexin, metronidazole), the patients recovered promptly and there were no complications. There were no clustering or outbreaks of endometritis in local community during the study period, and the endometritis cases were distributed evenly during the entire study period.

Neonatal infections, antibiotic treatment for newborns, and the need for neonatal intensive care unit treatment are shown in Table 4. There was no difference between the subgroups in terms of severe neonatal infections among the 1277 newborns, but there were more mild infections and a nonsignificant tendency for more antibiotic treatment in neonates whose mothers were Streptococcus B-positive.

## 4. Discussion

In this study, we found a clear association between the colonization of SDSE and postpartum endometritis. This is a novel observation, as there are very limited data published previously about the outcome of SDSE colonization among pregnant woman. In the past few years, there have been many articles that assume Streptococci G and C have a growing importance as clinically significant pathogens, but the association between SDSE colonization and puerperal endometritis has not yet been studied by other groups.

Prophylactic antimicrobial therapy during labor may have affected the incidence of postpartum infections in the groups. Streptococcus B*-*positive parturients were scheduled to be administered antibiotics, and most of the women were treated according to the guidelines. At least part of those delivering without antimicrobial treatment had a rapid birth, so there was not enough time to start antibiotic treatment prior to the birth. Prophylactic antimicrobial treatment, aimed at reducing the incidence of neonatal Streptococcus B infections, probably also diminished maternal infectious morbidity [17]. In the Streptococcus G or C group, more women received antimicrobial therapy during labor than those who had negative cultures. According to our estimation, besides antibiotics for suspected chorioamnionitis or prophylaxis during cesarean section, some 16% (67/421) of women incorrectly received prophylactic antibiotics in this group due to a misunderstanding of guidelines. In those cases, midwifes or doctors assumed that SDSE colonization in the swab culture also warrants prophylactic antibiotic treatment. It is noteworthy that postpartum endometritis was significantly more frequent in this group even if antimicrobial treatment during labor was more frequent. This may strengthen the conclusion that Streptococcus G or C is an important risk factor for postpartum infections.

There is still not enough evidence to recommend prophylactic antibiotic use for patients with SDSE colonization during labor. However, antibiotic treatment should start without delay for these women if other risk factors or signs of infection appear during labor or in the postpartum period. Because pregnancy alters the immune response, there is an increased risk for infections. In 2014, Acosta et al. reported on 365 cases of severe ante- and postpartum sepsis in the UK. The source of infection was the genital tract in 31% of cases, and 5.7% of infections were caused by Streptococcus G or C. 2.8% of the patients in that group had severe sepsis with septic shock [18]. Additionally, after the study period, we have had a few septic puerperal infections with a positive vaginal culture of Streptococcus G or C, including two cases of group G or C Streptococcus bacteremia. Both of these women had a long labor as an additional risk factor for puerperal infection. This strengthens the recognition of the potential pathogenicity of these bacteria. SDSE can no longer be considered simply a part of the normal microbiota; it is also an opportunist pathogen to pregnant woman.

We did not find a difference between the subgroups in severe neonatal infections (bacteremia or sepsis) in this study. Interestingly, in our data, the neonates of mothers colonized by Streptococcus B tended to receive more antibiotics than newborns in the other two groups, despite the fact that their mothers had already received antibiotic prophylaxis during the delivery. Although this was not a statistically significant finding, the result suggests that pediatricians are more likely to start antibiotics if the mother is known to be Streptococcus B-positive. Streptococcus B is the most common pathogen for early-onset neonatal sepsis, affecting 43–58% of cases, and the diagnosis of sepsis is challenging because many other noninfectious conditions resemble sepsis [19–21].

This study has some limitations. Some mild postpartum infections after delivery may have been treated at a primary care institution and as such could not be included in the study data, which was collated from hospital inpatient and outpatient material. Furthermore, it is possible that the colonization status had changed before the delivery, because in our protocol the rectovaginal swabs were obtained only once between the 35th and 37th weeks of gestation. However, with regard to previously published data, we can assume that this does not change our results significantly. In 2015, Kunze et al. found that the positive predictive value of a positive prepartum screening result for Streptococcus B positivity at delivery is over 77%. Their dataset included more than 900 patients, and the maternal Streptococcus B colonization rates were similar when comparing pre- and intrapartum screening [22]. A later timepoint for the swab could provide even more up-to-date knowledge of the colonization during the labor. On the other hand, late preterm or early term labors would happen without the knowledge of Streptococcus colonization, and that would increase antibiotic use among these parturients. In some units, an intrapartum group B Streptococcus screening is used in the labor ward. In a study by Plainvert et al., this method found 78% of the positive cases, where the Streptococcus B culture was taken on the labor ward [23].

We did not take rectovaginal swabs or other lochia cultures to confirm the diagnosis during the puerperal period. This is another weakness of our study. Thus, it is possible that the woman had also been colonized by some other bacteria.

Our study is the first to report the association of SDSE colonization and postpartum endometritis. A major strength of this study is the large dataset, which covers 421 Streptococcus G- or C-positive pregnancies. The subgroups were comparable. In the study population, the attendance rate for rectovaginal swab screening was approximately 95%, which makes our data more reliable. Le Doare et al. recently examined the Streptococcus B screening policies of 95 countries. The median coverage was 80%, and 58% of the countries used microbiological screening [24].

There is a distinct need for new studies to discover whether the colonization of SDSE requires intrapartum antibiotic prophylaxis. There were more postpartum infections in our study group, but there were no differences in neonatal infections. At least in cases with additional risk factors for puerperal infection, antimicrobial therapy should be commenced after cutting the umbilical cord, or even earlier during labor.

## 5. Conclusions

2.9% of pregnant women are colonized by Streptococcus G or C. Vaginal colonization is associated with increased risk of postpartum endometritis. More research is needed to clarify if antibiotic prophylaxis is reasonable for this group, but antibiotic treatment should start without delay for these women if signs of infection appear during labor or in the postpartum period.

## Figures and Tables

**Table 1:** Selected clinical characteristics and demographics of the study population. Numbers are percentages or means (min, max).

	Streptococcus G or C	Streptococcus B	Negative	*p*-value
Mothers	*n* = 421	*n* = 421	*n* = 421	
Infants	*n* = 425	*n* = 424	*n* = 428	
Variables				
Age, years, mean (min, max)	30.3 (19,44)	30.3 (18,44)	30.2 (19,44)	0.973
BMI, median, kg/m^2^ (min, max)	23.5 (16,48)	23.5 (16,56)	22.9 (16,51)	0.114
BMI ≥ 30	16.2	18.6	18.1	0.051
Nulliparous	42.0	41.3	40.6	0.920
Current smoker	14.7	15.6	17.0	0.659
Caucasian	98.6	96.7	97.1	0.170
Gestational diabetes	16.7	19.2	17.8	0.496
Caesarean delivery	11.4	16.4	12.4	0.086
Anaemia	4.3	4.8	4.3	0.928
Antibiotic treatment during delivery^*∗*^	35.6	92.4	20.4	<0.001
Blood loss ≥ 1000 ml	5.9	6.9	7.4	0.675
Gestational age ≤ 37 weeks	4.8	2.9	5.7	0.115
Gestational age ≥ 42 weeks	6.2	3.1	2.1	0.007
Apgar score 1 min < 3	0.5	1.2	0.9	0.527
Apgar score 5 min < 7	1.6	1.9	2.3	0.795

BMI = body mass index (calculated as persons weight in kilograms divided by square of height in meters). ^*∗*^Including prophylactic antibiotics for Streptococcus B colonization, treatment for suspected chorioamnionitis, and prophylactic antibiotics during cesarean section. Age and BMI were compared between three groups using ANOVA or the Kruskall-Wallis test and the other variables with chi-squared test or Fisher's exact test.

**Table 2:** Postpartum maternal infections in the study groups.

Infection	Streptococcus G or C	Streptococcus B	Negative	*p* value
	*n* (%)	*n* (%)	*n* (%)	
Endometritis	18 (4.2)	3 (0.7)	1 (0.2)	<0.001
Urinary tract infection	2 (0.5)	1 (0.2)	1 (0.2)	1.00
Wound infection	3 (0.7)	2 (0.5)	2 (0.5)	1.00
Bacteremia	1 (0.2)	1 (0.2)	0 (0.0)	1.00
Any infection	24 (5.7)	7 (1.7)	4 (1.0)	<0.001

Differences between three study groups were assessed using Fishers exact test.

**Table 3:** Case by case analysis of the Streptococcus G or C positive patients with endometritis.

Patient	Mode of delivery	Gestational age	Days from delivery to first symptoms	Leukocyte (max.)	CRP (max.)	i.v antibiotics^*∗*^	p.o antibiotics^*∗*^	Infant
1	vaginal	42+1	10	13.1	59.5	-	7–10	No signs of infection. CRP 4.7, leuk 16.
2	vaginal	41+1	12	15.3	8.0	-	7–10	No signs of infection. CRP 3.3, leuk 12.7.
3	vaginal	37+3	5	18	225	5	7	No signs of infection. No laboratory tests.
4	acute CS	35+5	6	16.4	267	8	7	No signs of infection. No laboratory tests.
5	vaginal	38+2	7	17.4	11.8	-	7–10	No signs of infection. No laboratory tests.
6	vaginal	40+0	12	5.9	13.7	-	7–10	No signs of infection. CRP 2.7, leuk 13.3.
7	vaginal	39+6	3	10.8	173	2	7	No signs of infection. No laboratory tests.
8	planned CS twins	37+2	20	3.3	234	3	7	A: NICU 9 days, iv. G-pen + Tomycin. CRP 39, Neutropenia.
								B: NICU 15 days, iv G-pen + Tomycin. CRP 36, Neutropenia.
9	vaginal	40+5	3	13.0	94.9	3	7	No signs of infection. CRP 9.2, leuk 17.6.
10	vaginal	39+4	5	13.0	70.5	5	7	No signs of infection. CRP < 1, leuk 11.7.
11	vaginal	39+6	5	14.7	102	3	7	No signs of infection. No laboratory tests.
12	vaginal	41+0	3	14.8	146	2	10	No signs of infection. No laboratory tests.
13	vaginal	39+3	4	8.6	182.9	3	7	No signs of infection. No laboratory tests.
14	vaginal	41+0	4	14.2	159	4	7	No signs of infection. CRP 5.1, leuk 22.6.
15	vaginal	39+3	4	14	96.7	4	7	No signs of infection. No laboratory tests.
16	vaginal	35+4	12	8.9	56	-	7–10	No signs of infection. CRP 1.6, leuk 18.4.
17	vaginal	40+4	4	16.1	146	-	7–10	No signs of infection. CRP 3.1, leuk 21.4.
18	vaginal	37+1	4	16.9	147	3	7	No signs of infection. No laboratory tests.

^*∗*^Duration (days). I.v. antibiotics = intravenous antibiotic treatment, usually cefuroxime 1.5 g and metronidazole 500 mg three times per day. P.o. antibiotics = orally antibiotic treatment, usually cephalexin 500 mg and metronidazole 500 mg three times per day. CS, cesarean section; CRP, C-reactive protein; leuk, leukocyte; NICU, neonatal intensive care unit.

**Table 4:** Neonatal infections and antibiotic and intensive care treatment in the study groups.

	Streptococcus G or C	Streptococcus B	Negative	*p*value
	*n* (%)	*n* (%)	*n* (%)	
Bacteremia, sepsis	0 (0)	0 (0)	1 (0.2)	1.00
Other infections^*∗*^	28 (6.6)	40 (9.5)	19 (4.5)	0.015
Antibiotic treatment	35 (8.3)	45 (10.7)	28 (6.7)	0.103
NICU	40 (9.5)	51 (12.1)	38 (9.0)	0.272

^*∗*^Other infections are skin, urinary tract, navel infections, or suspicion of infections; NICU, neonatal intensive care unit; differences between three study groups were assessed using Fisher's exact test.

## Data Availability

The data used to support the findings of this study are available from the corresponding author upon request.

## Abbreviations

SDSE: * Streptococcus dysgalactiae subspecies equisimilis*, Streptococcus group C or G
SDSD: * Streptococcus dysgalactiae subspecies dysgalactiae*
STSS: Streptococcal toxic shock syndrome
GBS: Group B Streptococci
BMI: Body mass index
NICU: Neonatal intensive care unit.
